# Effectiveness of a play-based distraction technique in reducing gag reflex during intraoral periapical radiography in children: a randomised crossover trial

**DOI:** 10.1007/s40368-026-01186-y

**Published:** 2026-02-20

**Authors:** I. Singh, K. Sethia, Y. M. Karuna, N. Srikant, A. Rao, P. A. Nayak

**Affiliations:** Manipal College of dental Sciences Mangalore, Manipal Academy of Higher Education, Manipal, India

**Keywords:** Paediatric dentistry, Radiographs, Gagging, Distraction technique

## Abstract

**Purpose:**

Excessive gagging in children can hinder the successful completion of intraoral radiographs, adversely impacting diagnosis and treatment. The literature on the use of active distraction during intraoral radiographic techniques in children is limited. The study examined the relationship between the gag reflex and dental anxiety in paediatric patients as well as effectiveness of using an acrylic thumb light in reducing gag severity during intraoral periapical radiography.

**Methods:**

This in vivo comparative crossover study included 25 children aged 6–13 years with moderate to very severe gag reflex (classification of the gagging problem index). Each participant underwent intraoral periapical radiography twice: control (without distraction) and interventional (with play-based distraction using an acrylic thumb light), the order of which was randomised. Gag severity was assessed via the gagging severity (GS) criteria, and dental anxiety was measured via the Modified Child Dental Anxiety Scale–Faces version (MCDASf). The data were analysed via McNemar’s test and the Chi-square test.

**Results:**

A significant reduction in severe/worst gagging was observed in the intervention group compared with the control group (*p* = 0.001). Mild/moderate gag responses were noted in 84% of the children in the intervention group versus 40% in the control group. A significant association was found between higher MCDASf scores and increased gag severity in both the control (*p* < 0.001) and intervention (*p* = 0.019) conditions.

**Conclusion:**

Play-based distraction using an acrylic thumb light significantly reduced gag reflex severity during intraoral periapical radiography, with dental anxiety influencing gag response.

## Introduction

Dental treatments often elicit a particularly common and disruptive reaction known as the gag reflex among young patients (Eachempati et al. 2019). Gagging is often interpreted as a self-protective mechanism against perceived discomfort or invasion into the oral cavity (Sivakumar and Prabhu 2025). It is not uncommon for children to express increased gagging tendencies in response to the presence of dental instruments or the requirement of holding intraoral films in place while taking radiographs. The presence of the gag reflex can become an obstacle to completing routine dental procedures, including intraoral radiographic imaging, hindering the ability to obtain clear and accurate radiographic images necessary for diagnosis and treatment planning (Katsouda et al. 2021; Athanasiadou et al. 2025). This involuntary reaction can also increase a child’s distress in a dental setup (Eachempati et al. 2019). The literature shows a close association between dental anxiety and the gag reflex (Akarslan et al. 2013; Uziel et al. 2024).

The challenge of overcoming the gag reflex in paediatric dentistry is multifaceted. Traditional approaches to mitigate the gag reflex, such as focused breathing, the use of topical anaesthetics, and verbal reassurances, have been found to be only moderately successful (Eachempati et al. 2019; O’Donald et al. 2025). Moreover, the literature has largely focused on the use of these techniques during dental impression-making procedures (Farrier et al. 2011), with comparatively little attention given to gag responses elicited during intraoral radiographic examinations. This underscores the need for alternative techniques that are more effective and better tolerated by young patients (O’Donald et al. 2025).

One such approach gaining attention in paediatric dentistry is the use of the distraction technique (Mehdizadeh et al. 2023). Asking the child to engage in simple tasks such as raising a leg, counting numbers in mind, or watching a video can serve as helpful distractions during gag-evoking dental procedures. Play-based distraction is a novel technique that enables active deviation of attention. Playing, a natural and enjoyable activity for children, is certainly a powerful tool for reducing stress and anxiety, thereby improving cooperation in dental settings (Alsibai et al. 2023). The rationale behind play-based distraction is that by engaging children in playful activities, their attention can be diverted from potentially distressing stimuli, thus helping them overcome the perception of discomfort and mitigating the gag reflex.

Although play-based distraction has been a promising technique in both paediatric medicine and dentistry (Alsibai et al. 2023; Drape and Greenshields 2020), its specific role in managing potential gagging while taking intraoral radiographs remains underexplored. Thus, the present study aimed to evaluate the efficiency of active distraction in the form of an acrylic thumb light in paediatric patients in reducing the gag reflex while taking intraoral periapical radiographs and to correlate the dental anxiety of the child with the gagging reflex. An acrylic thumb light is a lightweight, small, transparent, battery-operated LED device that can be worn over the thumb. It emits a soft, colored light upon pressing an embedded button. Thus, it provides play-based distraction, thereby drawing the patient’s attention away from the procedure when used during dental radiography. The null hypothesis for the study was that there would be no correlation between the child’s degree of dental anxiety and the gagging reflex and that there would be no difference in the gag reflex while taking intraoral periapical radiographs with or without active distraction.

## Methods

This in vivo comparative crossover randomised controlled study **(**registered in the Clinical Trials Registry- India, CTRI/2025/05/087760) was conducted in the Department of Paediatric and Preventive Dentistry, Department of Oral Medicine and Radiology, MCODS Mangalore, after approval was obtained from the Institutional Ethics Committee (reference no: 24057). The study was conducted under the Helsinki Declaration 2024 ethical principles for medical research involving human participants, following the Consolidated Standards of Reporting Trials (CONSORT) guidelines.

The study was conducted on children aged 6–13 years whose parents agreed to provide written consent. Children with a minimum requirement of two intraoral periapical radiographs either in the maxillary or mandibular molar region (both radiographic requirements in the same arch) for diagnosis or treatment planning, or as a part of the treatment, children with moderate to very severe gag reflex according to the Classification of Gagging Problem (CGP) index (Saita et al 2013) and children with Frankl’s definitely positive or positive behavior ratings were included. Uncooperative children (McDonald et al 2004), those who have special health care needs, were excluded. Children who were taking any kind of systemic medication during the study period, who needed emergency dental treatment, and who had a minimum of two intraoral periapical radiographic requirements but were not on the same arch were also excluded.

The sample size for this study was calculated based on the values derived from the study by Dixit and Moorthy (2021), who used the formula for calculating the sample size of two proportions. With a 5% alpha error, a power of 80% (1-β), corresponding to a Z(1-β) value of 0.84, and a calculated difference between the two proportions, p1–p2, of 0.36, the yielded sample size was 25 participants.

### Study setting and procedure

Children presenting to the Department of Paediatric and Preventive Dentistry, if they fulfilled the set inclusion and exclusion criteria, were assessed for their inherent gagging reflex via the CGP index (Saita et al 2013). The CGP index is a stepwise clinical method used to assess gag reflex severity based on patient tolerance to progressively invasive dental procedures. It was assessed during a clinical examination by a trained paediatric dentist, who conducted the examination in a dental chair using a dental mirror and a probe under standard conditions. The procedures included momentary mouth mirror insertion, anterior teeth examination via a mouth mirror, molar examination via a mirror, and basic periodontal probing. The gagging is classified into five grades: G1 (normal gagging but not desensitised), G2 (mild), G3 (moderate), G4 (severe), and G5 (very severe). The children found to have moderate to very severe gag reflexes were subsequently chosen for the study. The parents of the eligible participants were given an information summary to read, along with verbal explanations of the procedure. Written informed consent and assent (when applicable) were obtained.

The child’s dental anxiety level was measured via the Faces version of the Modified Child Dental Anxiety Scale (MCDASf) (Howard and Freeman 2007). The Faces version of the MCDASf is a child-friendly self-report questionnaire that consists of eight items related to common dental situations, each accompanied by a series of facial expressions ranging from very happy (no anxiety) to very distressed (high anxiety). Children are asked to select the face that best represents how they feel, with responses scored on a 5-point scale, where higher scores indicate greater dental anxiety. Although the facial format improved understanding and reliability in younger children, if the child was unable to comprehend the items in the questionnaire, he/she was allowed parental assistance. The total MCDASf score was calculated, and the anxiety of the child was recorded before being subjected to the radiographic technique.

The study consisted of two groups: 1) the control group and 2) the play-based distraction group (using an acrylic thumb light). As this was a crossover study, the same patient underwent both the control and experimental intraoral periapical radiographic procedures. The order of procedures was randomly determined by a coin flip: heads indicated the conventional procedure first, and tails indicated play-based distraction first. The washout period between the control and interventional radiographic procedures was not necessary, as the intervention was nonpharmacological and had an immediate effect.

**Fig. 1:**
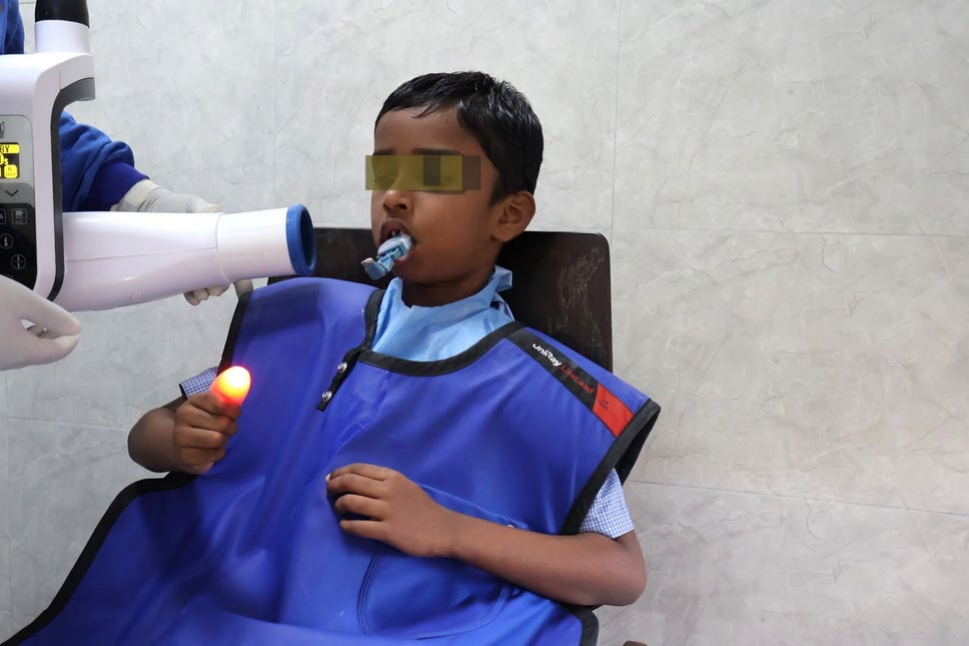
Taking an intraoral periapical radiograph while the child is playing with an acrylic thumb

The digital intraoral periapical radiographs were taken by an experienced paediatric dentist via photostimulable phosphor (PSP) plates of size #2, depending upon the patient’s arch size, and the bisecting angle technique was used. The PAP plates were exposed via an intraoral X-ray unit at 70 kVp, 8 mA, and an exposure time of 0.16 s (Ghom 2008). Radiation protection was ensured by using protective lead aprons for the patient and the operator. For both the control and play-based distraction groups, the procedures were described to the patients, and they were instructed to breathe deeply once and swallow before opening their mouths for film placement. The patients were asked to perform deep breathing through the nose while the radiograph was taken (Sewerin 1984). For the play-based distraction group, in addition to these basic instructions, the patients were shown how to use the acrylic thumb light (RS Magic tricks, Mumbai, India), which was placed on their thumb, and instructions were given to play with it during the radiographic technique (Fig. 1). Once the children learned to use it, they were asked to take a deep breath and open their mouths for film placement. Intraoral radiographs were taken, encouraging the patients to practice what had been instructed.

The severity of gagging during radiographic procedures was visually evaluated and scored using the gagging severity (GS) criteria (Sewerin 1984). Patients are categorised into one of the following categories depending on their gag severity. Category 0 indicates no or easily controlled mild gag reflex; Category 1 indicates moderate gagging that allows intraoral radiography with preventive measures; Category 2 indicates severe gagging requiring special precautions and multiple attempts to complete intraoral radiography; and Category 3 indicates an extreme gag reflex with vomiting or refusal, making intraoral radiography impossible.

All evaluations were conducted independently by two evaluators (paediatric dentists). Before data collection, both evaluators underwent standardised training sessions that included a detailed review of the assessment criteria and calibration exercises using pilot samples not included in the final analysis. In cases of interexaminer disagreement during the final analysis, a consensus was reached through discussion. Furthermore, interexaminer reliability was assessed via Cohen’s kappa coefficient, and it demonstrated good agreement between examiners (*κ* = 0.8). Evaluator blinding was not possible due to the visible nature of the intervention.

### Statistical analysis

The data analyst was blinded to the study. The collected data were analysed via SPSS software version 20.00 (SPSS, Chicago, IL). McNemar’s analysis was used to analyse the difference in paired categorical outcomes (gag severity) between the two treatment groups (control and intervention). A Chi-square test was used to analyse the association between the child’s dental anxiety and the severity of gagging in both the experimental and control groups. The level of significance was set at 0.05.

## Results

McNemar’s analysis, which is used to analyse the difference in paired categorical outcomes (gag severity) between the two treatments, revealed that the severe/worst gag scores were significantly lower and that the mild/moderate gag scores were substantially greater when the play-based distraction technique was used (*p* = 0.001) (Table 1). For the intergroup comparisons, the severe and worst categories were combined. This consolidation was performed to address sparse data in the extreme categories and to improve the robustness and interpretability of the statistical analysis.

**Table 1:** Cross-tabulation of the severity of gagging seen in the control group versus the play-based distraction group

Gagging severity	Control group *n* (%)	Play-based distraction group *n* (%)
Mild	0 (0.0)	14 (56.0)
Moderate	10 (40.0)	7 (28.0)
Severe/worst	15 (60.0)	4 (16.0)
Total	25 (100)	25 (100)

McNemar test, *p* = 0.001*

*statistically significant *p* value, binomial distribution used

When the association between the MCDASf score and the severity of gagging was analysed via the Chi-square test in both the control and the play-based distraction groups, a statistically significant association was observed (*p* = 0.00005 and *p* = 0.019, respectively). In the control, 85.7% of those with an MCDASf score of 5 experienced the worst gagging, whereas 75% with an MCDASf score of 4 experienced severe gagging (Table 2). In the play-based distraction group, 50% of the participants with an MCDASf score of 3 experienced nil/mild gagging, whereas 100% of those with a score of 5 experienced the worst gagging (Table 3).

**Table 2:** Association between MCDASf score and the severity of gagging as analysed using the Chi-square test in the control group

MCDASf Score	Severity of gagging (control group)				Total
	Mild *n* (%)	Moderate *n* (%)	Severe *n* (%)	Worst *n* (%)	
Score 1	0 (0.0%)	1 (10.0%)	0 (0.0%)	0 (0.0%)	1 (4.0%)
Score 2	0 (0.0%)	1 (10.0%)	0 (0.0%)	1 (14.3%)	2 (8.0%)
Score 3	0 (0.0%)	7 (70.0%)	2 (25.0%)	0 (0.0%)	9 (36.0%)
Score 4	0 (0.0%)	1 (10.0%)	6 (75.0%)	0 (0.0%)	7 (28.0%)
Score 5	0 (0.0%)	0 (0.0%)	0 (0.0%)	6 (85.7%)	6 (24.0%)
Total	0 (0.0%)	10 (100.0%)	8 (100.0%)	7 (100.0%)	25 (100.0%)

Pearson Chi-square = 33.393, df = 8, *p* = 0.00005

**Table 3:** Association between MCDASf score and the severity of gagging as analysed using the Chi-square test in the play-based distraction group

MCDASf score	Severity of gagging (play-based distraction group)				
	Nil/Mild *n* (%)	Moderate *n* (%)	Severe *n* (%)	Worst *n* (%)	Total
Score 1	1 (7.1%)	0 (0.0%)	0 (0.0%)	0 (0.0%)	1 (4.0%)
Score 2	1 (7.1%)	1 (14.3%)	0 (0.0%)	0 (0.0%)	2 (8.0%)
Score 3	7 (50.0%)	2 (28.6%)	0 (0.0%)	0 (0.0%)	9 (36.0%)
Score 4	5 (35.7%)	2 (28.6%)	0 (0.0%)	0 (0.0%)	7 (28.0%)
Score 5	0 (0.0%)	2 (28.6%)	0 (0.0%)	4 (100.0%)	6 (24.0%)
Total	14 (100.0%)	7 (100.0%)	0 (0.0%)	4 (100.0%)	25 (100.0%)

Pearson Chi-square = 18.240, df = 8, *p* = 0.019

## Discussion

The gag reflex is an involuntary protective reflex that protects the trachea, larynx, or pharynx from the sudden entry of foreign bodies (Kumar et al. 2011). In dentistry, patients with a high gag reflex present a significant clinical challenge in all domains, from diagnosis to treatment (Colvenkar et al. 2022). A study by Katsouda et al. (2021) revealed that 21% of 4- to 12 year-old children gag while taking dental radiographs, and the children who gagged during dental radiographs had significantly higher gagging assessment scale (GAS) scores. The present study used a play-based distraction technique as an attempt to decrease gagging during the intraoral periapical radiographic technique among the children found to have moderate to very severe gag reflexes when the CGP index was used. We used an acrylic thumb light as the mode of play-based distraction because of its simplicity, cost-effectiveness, non-invasive nature, and ease of use.

The use of play-based distraction appears to serve as an effective cognitive‒behavioural approach by redirecting the child’s focus from the invasive and uncomfortable stimulus of intraoral radiography, as the findings of this study revealed a significant reduction in the severity of gag reflexes following the intervention. These findings align with the existing literature that emphasises the benefits of nonpharmacological behaviour management strategies in pediatric dentistry, particularly those based on distraction and engagement. A study conducted by Linthoingambi et al. (2022) recommended the use of distraction methods such as intellectual colour games, audio–visual, and stress-ball techniques for the management of gagging and anxiety in children while recording dental impressions. Similarly, a study by Dixit and Moorthy (2021) concluded that a simple and non-invasive interactive distraction technique was effective in managing gagging while making maxillary impressions in 5- to 10 year-old children. However, these studies successfully used distraction techniques for the management of gagging while recording dental impressions, in contrast to the present study, which used a play-based distraction technique for the management of gagging during the intraoral periapical radiographic technique.

The positive relationship between children’s dental fear/anxiety and increased gagging has been reported by various authors, such as Uziel et al. (2024), Katsouda et al. (2021), and Gucyetmez Topal et al. (2023). The present study also revealed that during both the control and interventional radiographic techniques, the children with higher dental anxiety scores exhibited greater gagging sensations, thereby illuminating the psychological dimension of the underlying gag reflex. This finding reinforces the known relationship between dental anxiety and excessive gagging, highlighting the importance of addressing both psychological (dental anxiety) and physiological (gag reflex) factors for the effective dental management of paediatric patients.

Distraction is a very well-known method for effectively managing dental anxiety in children (Dahlan et al. 2023). For a technique to successfully act as a distracter from signals from unpleasant stimuli, it should involve multiple sensory modalities, allowing the patient to participate actively (Birnie et al. 2014). The play-based distraction technique used in our study, in the form of an acrylic thumb light, is one such method that keeps the child engaged owing to its simple yet attractive trick of illuminating light upon pressing. Thus, the intervention appeared to mitigate the gag reflex even among moderately anxious children, suggesting its broader applicability across varying anxiety levels. However, in a few children with very high anxiety (MCDASf score of 5), gagging persisted despite distraction, indicating that while play-based techniques are valuable, they may need to be supplemented with other behavior management techniques for optimal results.

According to the findings of our study, if a simple play-based distraction technique, such as an acrylic thumb light, is incorporated into routine radiographic procedures, it could not only improve patient experience and cooperation but also potentially reduce radiation risk by decreasing the need for repeated exposures. Additionally, it is a cost-effective and easily implementable strategy that aligns with child-centred care principles.

Despite these promising results, the study has certain limitations. Although mitigated through standard scoring criteria, the subjective nature of gag severity assessment performed in the present study might cause observer bias. Additionally, the relatively small sample size may impact the generalisability of the findings. Thus, future studies could benefit if objective physiological markers are incorporated along with the subjective assessment of gagging. Additionally, it would be more beneficial if the lasting impact of repeated exposure to play-based interventions and whether they lead to desensitisation over time were assessed through longitudinal studies.

## Conclusion

Within these limitations, the present study demonstrated that a play-based distraction technique in the form of an acrylic thumb light was a successful strategy to overcome gag reflexes in paediatric patients during intraoral periapical radiography. The study also highlighted the influence of dental anxiety on the severity of the gag reflex.

## Data Availability

The raw data and digital images used in the study are stored locally with the corresponding author and are freely available on request.

## References

[CR1] Akarslan ZZ, Yıldırım Biçer AZ. Influence of gag reflex on dental attendance, dental anxiety, self-reported temporomandibular disorders and prosthetic restorations. J Oral Rehabil. 2013;40(12):932–9. 10.1111/joor.12106. 24118087 10.1111/joor.12106

[CR2] Alsibai E, Bshara N, Alzoubi H, Alsabek L. Assessing an active distracting technique during primary mandibular molar pulpotomy (randomized controlled trial). Clin Exp Dent Res. 2023;9(2):283–9. 10.1002/cre2.702. 36478192 10.1002/cre2.702 PMC10098273

[CR3] Athanasiadou P, Arhakis A, Balli D, Zarkadi AE, Arapostathis K, Boka V. The impact of radiographic examination timing and gag reflex on dental fear and cooperation in children: a comparative clinical study. Eur Arch Paediatr Dent. 2025;26(2):375–83. 10.1007/s40368-024-00993-5. 39789394 10.1007/s40368-024-00993-5

[CR4] Birnie KA, Noel M, Parker JA, Chambers CT, Uman LS, Kisely SR, et al. Systematic review and meta-analysis of distraction and hypnosis for needle-related pain and distress in children and adolescents. J Pediatr Psychol. 2014;39(8):783–808. 10.1093/jpepsy/jsu029. 24891439 10.1093/jpepsy/jsu029 PMC4138805

[CR5] Colvenkar S, Reddy V, Thotapalli S, Deepa Rani K, Bharadwaj S. A simple step-by-step technique for the management of gagging in edentulous patient. Cureus. 2022;14(4):e24423. 10.7759/cureus.24423. 35637810 10.7759/cureus.24423 PMC9127034

[CR6] Dahlan M, Alsaywed R, Alamoudi R, Batarfi AA, Basodan OY, Gazzaz Y, et al. Assessment of different distraction behavioral methods in pediatric dental clinic: a systematic review. Cureus. 2023;15(7):e42366. 10.7759/cureus.42366. 37621781 10.7759/cureus.42366 PMC10445507

[CR7] Dixit UB, Moorthy L. The use of interactive distraction technique to manage gagging during impression taking in children: a single-blind, randomised controlled trial. Eur Arch Paediatr Dent. 2021;22(2):219–25. 10.1007/s40368-020-00582-2. 33247395 10.1007/s40368-020-00582-2

[CR8] Drape K, Greenshields S. Using play as a distraction technique for children undergoing medical procedures. Br J Nurs. 2020;29(3):142–3. 10.12968/bjon.2020.29.3.142. 32053441 10.12968/bjon.2020.29.3.142

[CR9] Eachempati P, Kumbargere Nagraj S, Kiran Kumar Krishanappa S, George RP, Soe HHK, Karanth L. Management of gag reflex for patients undergoing dental treatment. Cochrane Database Syst Rev. 2019;2019(11):CD011116. 10.1002/14651858.CD011116. 31721146 10.1002/14651858.CD011116.pub3 PMC6953338

[CR10] Farrier S, Pretty IA, Lynch CD, Addy LD. Gagging during impression making: techniques for reduction. Dent Update. 2011;38(3):171–2, 174-6. 10.12968/denu.2011.38.3.171. 21667831 10.12968/denu.2011.38.3.171

[CR11] Ghom A. Specialized intraoral techniques. In: Ghom AG, editor. Textbook of oral radiology. Logix Park: Elsevier Inc; 2008. p. 177–87.

[CR12] Gucyetmez Topal B, Falay Civelek SB, Tiras M, Yigit T. The prevalence and influencing factors of gag reflex in children aged 7-14 years in the dental setting. J Oral Rehabil. 2023;50(5):376–82. 10.1111/joor.13432. 36794577 10.1111/joor.13432

[CR13] Howard KE, Freeman R. Reliability and validity of a faces version of the Modified Child Dental Anxiety Scale. Int J Paediatr Dent. 2007;17(4):281–8. 10.1111/j.1365-263X.2007.00830.x. 17559456 10.1111/j.1365-263X.2007.00830.x

[CR14] Katsouda M, Coolidge T, Simos G, Kotsanos N, Arapostathis KN. Factors associated with gagging during radiographic and intraoral photographic examinations in 4-12-year-old children. Eur Arch Paediatr Dent. 2021;22(2):129–37. 10.1007/s40368-020-00535-9. 32440854 10.1007/s40368-020-00535-9

[CR15] Kumar S, Satheesh P, Savadi RC. Gagging. N Y State Dent J. 2011;77(4):22–7 (**PMID: 21894827**). 21894827

[CR16] Linthoingambi A, Harsimran K, Rishika C, Ramakrishna Y. Effectiveness of intellectual color game, audio-visual and stress ball distraction methods on gagging and anxiety management in children. J Clin Pediatr Dent. 2022;46(6):6–10. 10.22514/jocpd.2022.019. 36624898 10.22514/jocpd.2022.019

[CR17] McDonald RE, Avery DR, Dean AJ. In: Dentistry for the child and adolescent. 8th ed. St. Louis: Mosby; 2004. p. 729.

[CR18] Mehdizadeh M, Mohammadbeigi A, Sharifinejad A. An overview about new methods in management of gag reflex during dental treatment: a systematic review. J Dent (Shiraz). 2023;24(4):372–81. 10.30476/dentjods.2022.96360.1934. 38149230 10.30476/dentjods.2022.96360.1934 PMC10749434

[CR19] O’Donald F, Smith M, Sevier-Guy LJ, Heffernan A. Psychological interventions for gagging: implications for dental practice. Spec Care Dentist. 2025;45(1):e13090. 10.1111/scd.13090. 39791525 10.1111/scd.13090 PMC11720398

[CR20] Saita N, Fukuda K, Koukita Y, Ichinohe T, Yamashita S. Relationship between gagging severity and its management in dentistry. J Oral Rehabil. 2013;40(2):106–11. 10.1111/joor.12014. 23231041 10.1111/joor.12014

[CR21] Sewerin I. Gagging in dental radiography. Oral Surg Oral Med Oral Pathol. 1984;58(6):725–8. 10.1016/0030-4220(84)90043-4. 6594663 10.1016/0030-4220(84)90043-4

[CR22] Sivakumar S, Prabhu A. Physiology, gag reflex. In: StatPearls. Treasure Island: StatPearls Publishing; 2025. 32119389

[CR23] Uziel N, Gilon E, Bar I, Edri N, Eli I. Excessive gag reflex, dental anxiety, and phobia of vomiting in dental care. Int Dent J. 2024;74(4):801–7. 10.1016/j.identj.2023.12.002. 38228431 10.1016/j.identj.2023.12.002 PMC11287088

